# Leukocyte-Rich Platelet-Rich Plasma as an Effective Source of Molecules That Modulate Local Immune and Inflammatory Cell Responses

**DOI:** 10.1155/2022/8059622

**Published:** 2022-08-02

**Authors:** Maciej Dejnek, Helena Moreira, Sylwia Płaczkowska, Ewa Barg, Paweł Reichert, Aleksandra Królikowska

**Affiliations:** ^1^Clinical Department of Trauma and Hand Surgery, Department of Trauma Surgery, Faculty of Medicine, Wroclaw Medical University, 50-556 Wroclaw, Poland; ^2^Department of Medical Science Foundation, Faculty of Pharmacy, Wroclaw Medical University, 50-556, Wroclaw, Poland; ^3^Teaching and Research Diagnostic Laboratory, Department of Laboratory Diagnostics, Faculty of Pharmacy, Wroclaw Medical University, 50-556 Wroclaw, Poland; ^4^Ergonomics and Biomedical Monitoring Laboratory, Department of Physiotherapy, Faculty of Health Sciences, Wroclaw Medical University, 50-355 Wroclaw, Poland

## Abstract

Autologous platelet-rich plasma (PRP) injection is a safe biological method used to treat various musculoskeletal diseases. By downregulation of inflammatory cytokines and stimulation of synovial fibroblasts, PRP injection is a promising adjunctive treatment for patients with chronic autoimmune inflammatory diseases such as rheumatoid arthritis. A major problem in comparing the results of clinical trials in this area is the considerable variability in the cytokine content of PRP. We presented the profile of selected growth factors and inflammatory cytokines in the obtained PRP samples and compared them with baseline serum levels to assess the efficacy of PRP as a source of those paracrine molecules. Additionally, we wanted to determine whether the difference is only quantitative, which would suggest the use of a cheaper alternative by injecting a large amount of autologous serum. For this purpose, we analyzed whole blood and PRP samples prepared using the Mini GPS III Platelet Concentration System (Biomet Inc., USA) in 31 subjects aged 35-60 years. Cellular content, seven selected growth factors, and 13 human inflammatory cytokines were evaluated. Multiplex bead immunoassays that use fluorescence-encoded beads LEGENDplex™ (BioLegend, USA) and flow cytometer measurements were used. As a result, we found a statistically significant increase in four of the growth factors tested and eight of the inflammatory cytokines tested in PRP compared to blood serum. The difference is not only quantitative but also in the composition of paracrine molecules. In conclusion, the study confirmed that PRP is an efficient source of several growth factors and some inflammatory cytokines. These data provide additional insight into the potential mechanisms of PRP's effects on cellular metabolism and inflammatory response and may contribute to a better understanding of its clinical efficacy.

## 1. Introduction

Autologous platelet-rich plasma (PRP) injection is a treatment method used in various soft tissue degenerative conditions. It is widely used in orthopedic, sports medicine, stomatology, and aesthetic medicine [1–3]. A growing number of reports indicate a potential role for PRP in the treatment of involved joints in patients with chronic autoimmune inflammatory diseases such as rheumatoid arthritis [4, 5].

Rheumatoid arthritis (RA) is a chronic autoimmune inflammatory disease that affects approximately 1% of the world's population and is characterized by synovial hyperplasia, articular inflammation, and invasion of the synovium into the adjacent bone and cartilage [6]. The underlying cause of the disease is an abnormal immune response that activates fibroblast-like synoviocytes (FLS), provoking an inflammatory response that leads to disease progression [4]. In the initial stage, the disease usually affects small joints, but when advanced, it can lead to massive destruction of large joints such as the knee, shoulder, or hip, causing significant disability. The exact cause of the disease is not sufficiently understood, although many different therapies targeting the molecular pathways of the ongoing inflammatory process have been proposed [4]. One of the new methods supporting the treatment of patients is the intra-articular injection of PRP. Studies have shown high safety of the therapy resulting in suppression of the inflammatory process [4, 5, 7].

PRP is obtained by the separation of plasma with an excessive amount of platelets from the blood sample. The most common preparation method is manually extracting platelet-rich plasma from the blood sample after centrifugation in a specially designed tube [8]. Many different commercially available kits existing on the market are helping to prepare PRP as easily as possible in ambulatory conditions.

This treatment is aimed at providing a high amount of platelet-derived growth factors to enhance healing issues and stimulate degenerative tissue to regenerate or modulate the local inflammatory response [9]. In the human body, high amounts of growth factors and cytokines are released from platelets alpha-granules at the site of injury. Their role is to stimulate cell migration, proliferation, differentiation, angiogenesis, extracellular matrix production, and scar formation [10]. An enormous amount of various cytokines affect the local environment and interact with each other through the processes of stimulation and inhibition [9]. The function of many of them remains unclear and is difficult to assess in the living organism.

There are many doubts about the effectiveness of PRP treatment in different conditions. The results presented by various researchers are often mutually exclusive, and most meta-analyses do not provide clear answers in this field [1]. There may be several reasons for this situation. Significant differences in the content of biologically active components in PRPs prepared by different commercially available kits and their repeatability in the platelet and leukocyte concentrations are among the problems to be solved [11]. Furthermore, significant differences due to the patients' characteristics, such as age, sex, smoking status, diseases, drugs usage, or physical activity, can affect the quality of the final PRP product [12–14]. Incorrect interpretation of the results may also be due to the incompletely understood function of all cytokines in the PRP samples and their interaction with each other and the surrounding tissues. Many more studies are still needed both on the content of biologically active components in PRP samples and their influence on the effectiveness of treatment of various diseases.

The aim of the study was to assess if PRP samples would have a significantly higher amount of selected growth factors and inflammatory cytokines than baseline level in patients' serum and if the difference would be only quantitative or qualitative as well. We hypothesized that PRP would contain a proportionally higher concentration of paracrine molecules than patients' serum. Confirmation of the above hypothesis could lead to the development of a new, cheaper orthobiological treatment method by intra-articular injection of a higher volume of autologous serum, which could be beneficial for large joints affected by rheumatoid arthritis.

## 2. Materials and Methods

### 2.1. Ethical Standards

The study was carried out according to the Declaration of Helsinki and was approved by the Institutional Ethics Committee of Wroclaw Medical University (KB–26/2019, 21.01.2019). All patients agreed to participate in the study and signed an informed consent.

### 2.2. Study Design

The study was designed as a single-center prospective descriptive laboratory study and is an additional part of the investigation of the correlation between the concentration of cytokines in PRP and the effectiveness of epicondylopathy treatment registered in clinicaltrials.gov under identifier NCT04521387.

### 2.3. Population

The study was conducted on 31 patients who were enrolled in the years 2021-2022. The population contained 15 men and 16 women aged 35-60 years old (*x* = 49.10; SD = 6.03). Patients' height and weight were measured to calculate Body Mass Index (BMI). Three patients regularly smoked cigarettes, 15 regularly practiced sports activity (≥3 sessions per week), 22 drank alcohol occasionally (≤1 dose per week), and nine did not drink alcohol at all. Patients with hematologic diseases, diabetes, and suspicion of the infectious process; those who are pregnant; and those taking medications that may affect platelet function or the coagulation system were excluded.

### 2.4. PRP Preparation

**Figure 1:**
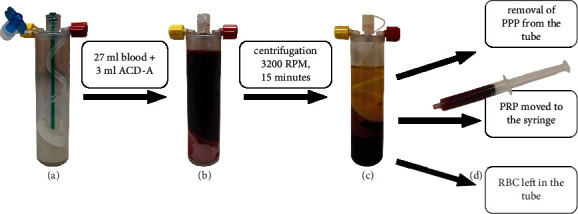
PRP preparation process by Mini GPS III. (a) The empty Mini GPS III tube. (b) The tube filled with 3 ml of anticoagulant citrate dextrose solution A (ACD-A) and 27 ml of patients' own blood. (c) The tube after centrifugation containing three separate layers: platelet-poor plasma (PPP), platelet-rich plasma (PRP), and red blood cells (RBC). (d) The syringe filled with PRP taken from the tube after the removal of PPP.

From each patient, 27 ml of blood was collected into a 30 ml syringe filled with 3 ml of anticoagulant citrate dextrose solution A (ACD-A). Additional 6 ml of blood was taken and divided to a 2 ml probe with ethylenediaminetetraacetic acid (EDTA) for complete blood count analysis and a 4 ml probe with clotting activator for serum preparation. A 30 ml syringe blood sample mixed with ACD-A was transferred to a specially designed tube with a membrane for PRP separation—Mini GPS III Platelet Concentration System (Biomet Inc., USA). The tube was placed in a dedicated centrifuge for the separation process, which took 15 minutes with 3200 revolutions per minute (RPM). After centrifugation, platelet-poor plasma placed above the separation membrane was removed, and 3 ml liquid-form leukocyte-rich platelet-rich plasma was collected in the sterile 3 ml syringe according to the manufacturer manual. The step-by-step process for preparing PRP is shown in Figure 1. Two ml of PRP was used for patient treatment, and 1 ml of PRP was reinjected into an Eppendorf polypropylene tube and then gently shaken for 30 seconds just before laboratory analysis. The time between blood draw, PRP separation, and further analysis did not exceed 1 hour, and the whole process was conducted in daylight at room temperature.

### 2.5. Evaluation of Cellular Components

The complete blood count was analyzed from a 2 ml probe with EDTA using the Mindray BC-5150 automatic laboratory analyzer (Shenzhen Mindray Bio-Medical Electronics Co., PRC). Then, the PRP sample was analyzed in the same fashion from the Eppendorf tube.

### 2.6. Serum Sample Preparation and Storage

Blood was collected into BD Vacutainer® Plus Plastic Serum Tubes (BD, Biosciences, Warsaw, Poland). The blood samples were allowed to clot at room temperature for approximately 20–30 min and centrifuged at 500 × g for 15 minutes. The serum was transferred to a polypropylene tube and stored at -80°C until the time of analysis.

### 2.7. PRP Activation and Storage

PRP samples were activated through a double freeze-thaw process for 30 minutes in each step as proposed by Zimmermann et al. [15]. The activated samples were frozen to -80°C and stored for further analysis.

### 2.8. Growth Factor and Inflammatory Cytokine Evaluation

LEGENDplex™ Custom Human 7-plex Panel and LEGENDplex™ Human Inflammation Panel 1 (BioLegend, USA) were used to estimate the concentration of selected growth factors and inflammatory cytokines in PRP and serum samples. The first one is a customized panel dedicated to our study, containing the most frequently studied and the most important platelet-derived growth factors. The second one is a commercially available standard set for inflammatory cytokine testing (see Table 1). LEGENDplex is a multiplex immunoassays based on fluorescence-encoded beads and flow cytometric measurements. Just before the assay, all samples were thawed to room temperature, centrifuged for 5 minutes at 2500 RPM in a Micro Star 17 microcentrifuge (VWR International Company, Thermo Electron LED, Germany), and 2x diluted. A LEGENDplex assay was performed according to the manufacturer's procedure. The samples were acquired on a CyFlow Cube 8 flow cytometer (Sysmex-Partec, Gӧrlitz, Germany) applying a 488 nm laser with 536/40 (BP) filter for the PE fluorochrome and a 638 nm laser with 675/20 (BP) for the APC fluorochrome. The results were analyzed using LEGENDplex™ Data Analysis Software V.8.0 (Vigene Tech Inc., USA). The concentration of each growth factor/cytokine was determined by means of a standard curve generated during the performance of the assay.

### 2.9. Statistical Analysis

For statistical assessment, Statistica 13.3 software (TIBCO Software Inc, USA) was used. For compliance of the result distribution, the Shapiro-Wilk test was used. Arithmetic means and standard deviations (SD) were calculated. For data with nonnormal distribution, the median and quartile distribution (Q1-Q3) were additionally given. The outliers (more than 3 standard deviations) were removed for the calculations. For comparison of two variables with normal distribution, dependent *t*-test for paired samples was performed. For variables without normal distribution, the Wilcoxon signed rank test was performed. Pearson's correlation coefficient (*r*) was used to establish a relationship between blood cell components and growth factors or inflammatory cytokines. The level equal to or greater than 0.8 was assumed to be the satisfactory power of the tests (1 − *β* > 0.8). Based on an earlier pilot study, we calculated that 30 patients would be sufficient to achieve the power target for the growth factor comparison [11]. The results were assessed as significant at *p* ≤ 0.05.

## 3. Results

### 3.1. Cellular Components

Differences between whole blood and PRP samples in cellular content are highlighted in Table 2. All differences except the content of eosinophils (*p* = 0.52) were significant (*p* ≤ 0.001). WBC and PLT but not RBC content in whole blood correlate (*r* > 0.30) to their concentrations in PRP. There was significant difference between men and women in RBC content in whole blood (5.15 *vs.* 4.56, *p* ≤ 0.001), which is a physiological difference within the normal range for both sexes. WBC content in PRP also differed between males and females (34.64 *vs.* 25.83, *p* < 0.05). Current smoking status was connected with a significantly higher concentration of PLT in whole blood and PRP (*p* < 0.05). Alcohol consumption and sports activity did not influence the cellular content of the whole blood and PRP.

### 3.2. Growth Factors

The differences between the serum and PRP growth factor content are presented in Table 3. All growth factors increased in PRP, but the difference was not significant for VEGF. Sex did not influence any of the growth factors. Smokers had significantly higher concentration of PDGF-BB (70486.93 *vs.* 46856.9, *p* < 0.05) which could be explained by a higher PLT content in their whole blood and PRP samples. Alcohol consumption and sports activity did not influence growth factor content in the whole blood and PRP.

### 3.3. Inflammatory Cytokines

Differences between serum and PRP inflammatory cytokine content are highlighted in Table 4. All inflammatory cytokine concentrations in the PRP were lower in women, and these differences were significant (*p* < 0.05) in all except MCP-1 (*p* = 0.63), IFN-*γ* (*p* = 0.10), TNF-*α* (*p* = 0.12), and IL-6 (*p* = 0.27). Comparison between both sexes is shown in Supplementary Table 1. Smoking status, alcohol consumption, and sports activity did not influence the content of cytokines in the PRP samples. More than 80% of the IL-23 results were below the lower cut-off point for measurement; therefore, it was excluded.

### 3.4. Correlations between Cell Content and Growth Factors or Inflammatory Cytokines

**Figure 2:**
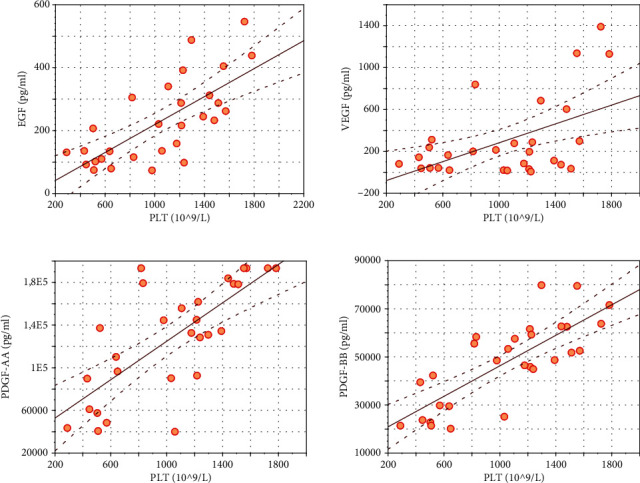
Significant (*p* < 0.05) Pearson correlation coefficient (*r*) between platelets (PLT) and growth factors: EGF (*r* = 0.74), VEGF (*r* = 0.46), PDGF-AA (*r* = 0.77), and PDGF-BB (*r* = 0.79).

Significant high positive correlation was found between PLT content in PRP and three growth factors: EGF (*r* = 0.74; *p* ≤ 0.001), PDGF-AA (*r* = 0.77; *p* ≤ 0.001), and PDGF-BB (*r* = 0.79; *p* ≤ 0.001). Significant but low positive correlation was found between PLT and VEGF (*r* = 0.46; *p* < 0.05) (see Figure 2). No significant correlations between PLT content and inflammatory cytokines were observed.

**Figure 3:**
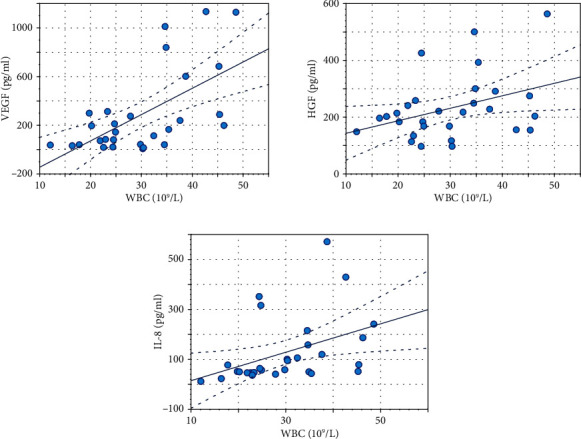
Significant (*p* < 0.05) Pearson correlation coefficient (*r*) between white blood cells (WBC) and growth factors/cytokines: VEGF (*r* = 0.69), HGF (*r* = 0.42), and IL-8 (*r* = 0.45).

Significant moderate positive correlation was found between WBC and VEGF (*r* = 0.69; *p* ≤ 0.001), and significant low correlation between WBC and HGF (*r* = 0.42; *p* < 0.05). For inflammatory cytokines, a significant positive low correlation was found between WBC and IL-8 (*r* = 0.45; *p* < 0.05) (see Figure 3). Subpopulation of neutrophils correlated moderately with IL-8 (*r* = 0.62; *p* ≤ 0.001) and on a low level with IL-1*β* (*r* = 0.41; *p* < 0.05) and IL-18 (*r* = 0.48; *p* < 0.05). Subpopulation of lymphocytes correlated moderately with FGF-basic (*r* = 0.53; *p* < 0.05) and on a low level with EGF (*r* = 0.43; *p* < 0.05) and VEGF (*r* = 0.48; *p* < 0.05).

Age and BMI did not influence the content of PLT, WBC, RBC, and all growth factors in PRP. Among inflammatory cytokines, age significantly negatively correlates with IL-1*β* (*r* = −0.50; *p* < 0.05), IFN-*α*2 (*r* = −0.38; *p* < 0.05), and TNF-*α* (*r* = −0.39; *p* < 0.05). BMI was positively correlated only with IL-1*β* (*r* = 0.40; *p* < 0.05). However, these correlations were on a low level.

## 4. Discussion

We hypothesized that PRP prepared with Mini GPS III Platelet Concentration System would contain a proportionally higher concentration of paracrine molecules than patients' serum. The results show that PRP delivers higher doses of TGF-*β*1 free active, EGF, VEGF, PDGF-AA, and PDGF-BB but not HGF and FGF-basic. However, the profile of inflammatory cytokines differed significantly only for eight from thirteen measured: IL-1*β*, IFN-*α*2, IFN-*γ*, TNF-*α*, IL-6, IL-8, IL-10, and IL-12p70. These differences cannot be simply explained by a higher concentration of platelets or white blood cells. Increases in platelet-derived growth factor concentration (PDGF-AA, PDGF-BB) and TGF-*β*1 were closest to the achieved increase in platelet concentration (×6.19, ×6.43, and 3.79, respectively, vs. ×4.41). The two other growth factors increased more than two times (VEGF × 2.34, EGF × 2.5) and the other two decreased. Those inflammatory cytokines, the concentration of which increased significantly, increased from 1.39 to 5.89. This leads to the assumption that in PRP, we obtain a different cytokine profile, not just a quantitative increase in concentration. There is a risk of bias due to the serum preparation protocol, which requires cloth formation. Attention should be paid to this critical detail, as it can directly impact the clinical effectiveness of the proposed treatment.

Multiple protocols have been developed for PRP preparation. Many commercially available techniques differ in the shape of the separator used; the amount of blood collected and PRP obtained; the amount, time, and force of centrifugation; and the separation technique. As a result, they differ in the concentration of platelets, leukocytes, and red cell contamination obtained. These differences significantly affect the content of biologically active molecules that may have a therapeutic effect [8, 11]. Different divisions have been proposed to facilitate the comparison of PRP treatment results. The most commonly used takes into account fibrin and leukocyte content, dividing PRP into four categories: leukocyte-rich or pure platelet-rich plasma (L-PRP, P-PRP) and leukocyte-rich (L-PRF) or pure platelet-rich fibrin (P-PRF) [16]. Additionally, it is worth distinguishing systems enabling high (5-9×) and low (2.5-3×) concentrations of platelets [17]. Currently, the most complex classification system was proposed by Lana et al. [18]. The system called MARSPILL includes interalia, specifying the number of platelets, white blood cells, red blood cells, spins, activation process, and image guidance during PRP administration. The above classification perfectly illustrates the complexity of assessing the effectiveness of different PRP treatments.

Most researchers define PRP as plasma with platelet concentrations above 1 million per microliter [19, 20]. Commercially available systems do not always meet this criterion. The US Food and Drug Administration (FDA) allows the term PRP to be applied to products that have platelet concentrations above 250,000 per microliter. In addition, Mazzucco et al.'s work concluded that platelet concentrations above 200,000 per microliter are sufficient for a therapeutic effect [21]. This can lead to confusion as this platelet count is within the normal range for whole blood, and it is hard to expect a higher density of active cytokines in it. In our work, we used the Mini GPS III Platelet Concentration System, one of the most widely used PRP preparation systems globally. It allows obtaining reproducible results with 4-5 times platelet density and higher than baseline concentration of white blood cells [11].

The WBC content of the obtained PRP can influence the concentration of various cytokines and modulate local immune and inflammatory responses. Both negative and positive effects of leukocyte-rich PRP on tissue healing have been reported in the literature. Threats arise from the potential catabolic effects of leukocytes on surrounding tissues through the release of proinflammatory cytokines and proteinases. The benefits of a high WBC content are associated with positive correlations with certain growth factors, the ability of leukocytes to modulate immune responses, antimicrobial properties, and reported favourable clinical outcomes [22–25].

Previous studies have shown correlations between some cytokines and cell contents in PRP, but only a few have compared their contents and those in whole blood [25, 26]. A significant positive correlation was found between PLT content in PRP and EGF, VEGF, PDGF-AA, and PDGF-BB in these studies. Magalon et al. evaluated the content of EGF, VEGF, TGF-*β*1, and PDGF-AB in PRP obtained with Mini GPS III. The PLT and WBC concentrations were similar to our findings, but the growth factor content was much higher for VEGF and EGF (×3.2 and ×9.3, respectively) than in our study. The concentration of the representative of PDGF family in their research (PDGF-AB) was much lower than that of PDGF-AA or PDGF-BB in our study [27]. The possible reason for these differences is the addition of calcium chloride for platelet activation. The double freeze-thaw process activates platelets in a more physiological way, which is why it was used in our study [15]. Similar to our finding, they presented a significant positive correlation between EGF, VEGF, PDGF, and PLT concentration in PRP. Contrary to our results, they also found a correlation between PLT and TGF-*β*1 [27]. The concentration of WBC in PRP correlates with the content of VEGF and EGF in their study. Our findings support only the correlation between WBC and VEGF. Another study reporting growth factor content in PRP obtained by GPS III was carried out by Castillo et al. [28]. The concentration of WBC in the final sample was similar to that presented in our study, but PLT mean concentration was almost two times lower. That could be due to the fact that they used a different version of the GPS III system, which requires approximately 55 ml of blood donation and other centrifugation parameters. They assessed the final concentrations of PDGF-AB, PDGF-BB, TGF-*β*1, and VEGF. In their results, PDGF-BB was two times lower, but VEGF 6 times higher than those found in our study. Just as a decreased PDGF level can be explained by a lower platelet content, the differences in other factors are difficult to explain. In the literature review, Oudelaar et al. collected seven studies presenting the content of PLT, WBC, PDGF-AB, VEGF, and TGF- *β*1 in PRP obtained with the GPS III system [8]. The contents of PLT and WBC were similar between the studies and comparable to our results. However, the content of growth factors varied considerably, especially TGF-*β*1. In most of the mentioned studies, the concentration of VEGF was higher than in our samples. Concentrations of TGF-*β*1 were much higher because we evaluated the concentration of TGF- *β*1 free active, whose levels are about 65 times lower than total TGF-*β*1 in serum [29].

Among the best described is the platelet-derived growth factor family (PDGF) role in the healing process both in vitro, in animal models, and in patients with wound healing disorders. It stimulates neutrophils, monocytes, and fibroblasts to migrate to the wound site and activates the latter mentioned to proliferate and produce an extracellular matrix [9]. The fibroblast growth factor (FGF) family has mitogenic activity and a positive effect on cell migration and differentiation and participates in cytoprotection during stress conditions [10]. Angiogenesis at the wound site is induced and stimulated by vascular endothelial growth factor (VEGF) [9]. Other growth factors such as hepatocyte growth factor (HGF), epidermal growth factor (EGF), and transforming growth factor-*β*1 (TGF-*β*1) have an impact on proper cell proliferation and differentiation during the healing process [9, 10, 30].

Among a wide spectrum of tested inflammatory cytokines, a significant positive correlation was found only between WBC and IL-8. This cytokine has some potential positive effects on healing and immunomodulation by its ability to stimulate reepithelialization and to attract neutrophils to the site of injury. In the literature, only a few authors have performed an analysis of inflammatory cytokines in PRP. The concentrations of IL-1*β* and matrix metalloproteinase-9 (MMP-9) in PRP were presented in two studies [25, 26]. The authors found that MMP-9 and IL-1*β* levels were much higher in leukocyte-rich PRP and were significantly correlated with neutrophil concentration [25]. Those two are catabolic cytokines known for their role in inflammation and matrix degradation. MMP-9 has been implicated as a predictor of improper healing [26]. We also found significant but low correlation between neutrophil concentration and IL-1*β*. The healing process is also modulated by a number of different cytokines that can stimulate migration of macrophages like monocyte chemoattractant protein (MCP), stimulate reepithelialization such as interleukin-8 (IL-8), inhibit inflammation and scar formation such as interleukin-10 (IL-10) [10]. A complex process of interaction between numerous anti- and proinflammatory cytokines regulates the course of tissue healing, leading in the most desirable case to recovery.

In our study, age and BMI did not influence the content of PLT, WBC, RBC, and all growth factors in PRP. Dragoo et al., in their study, found a significant negative correlation between age and PDGF-BB. The concentration of PDGF-BB in the PRP was higher in 18-30-year-old subpopulation; however, among the older subpopulations, the values were stable and oscillated about 31 ng/ml [31]. Among inflammatory cytokines in our research, age was significantly negatively correlated with IL-1*β*, IFN-*α*2, and TNF-*α*. The statistically significant differences in the cytokine content in PRP between the sexes are difficult to explain, especially since they were not shown when comparing the serum samples. A possible explanation would be the uneven distribution of cytokines during centrifugation, forced by other components. Solving this problem would require further, more detailed research.

Rheumatoid arthritis is a chronic autoimmune inflammatory disease characterized by progressive destruction of cartilage and bone with periods of acute exacerbation [32]. The correlation of cytokines such as TNF-*α*, IL-6, and IL-1*β* with the intensity of the disease has been demonstrated [32]. This is also the reason why TNF-*α* inhibitors were developed to treat RA. The increased level of VEGF is probably related to the stimulation of neovascularization during the ongoing inflammatory process. In an animal model of arthritis, Lippross et al. found that PRP injection leads to the reduction of IL-6, IL-1, IGF-1, and VEGF in cartilage and synovium. TNF-*α* did not change after injection of PRP [32]. Tong et al. presented results on a type II collagen-induced arthritis mouse model treated with PRP. The study reported a downregulation of the expression of IL-6, IL-8, IL-17A, IL-1*β*, TNF-*α*, receptor activator for nuclear factor-*κ*B, and IFN-*γ* in inflammatory tissue [7]. They also found that PRP can be beneficial due to decreased joint inflammation, cartilage destruction, bone damage, and increased joint tissue repair [7]. On the other hand, in their papers, both Yan et al. and Wang et al. highlighted the risk of rheumatoid arthritis fibroblast-like synoviocytes cell migration, invasion, and adhesion stimulated by MMP-1, whose expression was increased after PRP administration [6, 33].

Only two studies evaluating PRP efficacy on rheumatoid arthritis have been published. Badsha et al. presented results of PRP injection to the knee joint of four subjects. They reported a significant improvement in the disease activity score, reduced pain, and a decrease of joint inflammation during ultrasound examination [5]. Saif et al. evaluated the therapeutic effect of intra-articular PRP versus steroid in RA patients and their impact on inflammatory cytokines, local joint inflammation, disease activity, and quality of life. In this randomized controlled trial, 60 patients with RA were divided into two equal groups. Both groups showed improvements at 3 months after injection, but only in the PRP group this improvement lasted up to 6 months. Downregulating effects on inflammatory cytokines (IL-1*β*, TNF-*α*) with subsequent improvement of local joint inflammation, disease activity, and quality of life were presented in the PRP group. The authors concluded that PRP injections were a safe and valuable treatment option for RA patients [34].

These findings lead us to the hypothesis that intra-articular PRP injections could help patients with RA through two main mechanisms. The first is the positive role of growth factors such as PDGF, EGF, FGF, or TGF-*β*1 in stimulating healing, regeneration, and protecting articular cartilage. The second is the ability of PRP to downregulate the expression of major inflammatory cytokines such as IL-1*β*, IL-6, and TNF-*α* leading to a reduction in local inflammation. The exact molecular pathway of the PRP interaction will be challenging to determine due to the multitude of biologically active ingredients it contains.

There are some limitations of the presented study. The major limitation is that we evaluated only one particular PRP preparation protocol, which, due to its distinctiveness, may in itself influence the final cytokine profile. Some of the existing numerous cytokines that may affect the local cellular response have been omitted for technical reasons. The direction of further research should be to compare the biological effect in vitro or in vivo depending on the profile of biologically active components in PRP.

## 5. Conclusions

The study showed that autologous leukocyte-rich platelet-rich plasma obtained with the Mini GPS III Platelet Concentration System is an efficient source of paracrine molecules such as TGF-*β*1, EGF, PDGF-AA, PDGF-BB, IL-1*β*, IFN-*α*2, TNF-*α*, and IL-8 with the ability to concentrate those molecules above twice as baseline. The profile of growth factors and cytokines is different in PRP than in patients' own blood serum. For the above reason, PRP cannot simply be replaced by an increased serum volume, which may, however, be considered for selected cases as an alternative treatment option.

## Figures and Tables

**Table 1:** All paracrine molecules tested by flow cytometry with the use of LEGENDplex™ panels.

LEGENDplex™ Custom Human 7-plex Panel	LEGENDplex™ Human Inflammation Panel 1
Transforming growth factor-*β*1 (TGF-*β*1, free active)	Interleukin-1*β* (IL-1*β*)
Epidermal growth factor (EGF)	Interferon-*α*2 (IFN-*α*2)
Fibroblast growth factor-basic (FGF-basic)	Interferon-*γ* (IFN-*γ*)
Vascular endothelial growth factor (VEGF)	Tumor necrosis factor *α* (TNF-*α*)
Hepatocyte growth factor (HGF)	Monocyte chemoattractant Protein-1 (MCP-1)
Platelet-derived growth factor-AA (PDGF-AA)	Interleukin-6 (IL-6)
Platelet-derived growth factor-BB (PDGF-BB)	Interleukin-8 (IL-8)
	Interleukin-10 (IL-10)
	Interleukin-12p70 (IL-12p70)
	Interleukin-17A (IL-17A)
	Interleukin-18 (IL-18)
	Interleukin-23 (IL-23)
	Interleukin-33 (IL-33)

**Table 2:** Differences in cellular content between whole blood (WB) and platelet-rich plasma (PRP). Values are presented as arithmetic mean (standard deviation). The ability to concentrate the cell components in PRP vs. WB is presented as the “ratio.” The Pearson correlation coefficient between the cell components of WB and PRP is presented as a value *r* (^∗^*p* < 0.050). The significance of the comparison is shown as *p* value.

	WB	PRP	Ratio	*p*	*r*
WBC (10^3^/*μ*l)	6.60 (1.34)	30.09 (9.58)	×4.59	≤0.001	0.66^∗^
Neutrophils	4.03 (1.22)	12.72 (6.66)	×3.19	≤0.001	0.47^∗^
Lymphocytes	1.96 (0.51)	14.18 (5.04)	×7.31	≤0.001	0.64^∗^
Monocytes	0.4 (0.1)	2.81 (1.07)	×7.25	≤0.001	0.73^∗^
Eosinophils	0.15 (0.11)	0.17 (0.17)	×1.06	0.52	0.78^∗^
Basophiles	0.03 (0.02)	0.19 (0.12)	×6.44	≤0.001	0.85^∗^
RBC (10^6^/*μ*l)	4.85 (0.44)	0.92 (0.49)	×0.19	≤0.001	0.14
PLT (10^3^/*μ*l)	253.27 (59.37)	1083.87 (493.55)	×4.41	≤0.001	0.47^∗^

**Table 3:** Differences in growth factor content between serum and platelet-rich plasma (PRP). Values (pg/ml) are presented as arithmetic mean (standard deviation) and median (Q1-Q3). The ability to concentrate growth factors in PRP vs. serum is presented as the “ratio.” The significance of the comparison is shown as *p* value.

	Serum		PRP		Ratio	*p*
	Mean (SD)	Median (Q1-Q3)	Mean (SD)	Median (Q1-Q3)		
TGF-*β*1, free active	151.67 (72.41)	158.02 (94.86-204.24)	383.33 (251.75)	343.26 (257.65-443.32)	×3.79	≤0.001
EGF	110.04 (44.88)	103.575 (76.33-140.36)	243.96 (155.21)	219.13 (116.43-319.81)	×2.5	≤0.001
FGF-basic	1094.06 (487.25)	1048.33 (667.71-1436.06)	746.30 (2043.42)	315.30 (254.70-428.24)	×0.68	≤0.001
VEGF	152.88 (52.91)	152.925 (128.87-171.73)	324.53 (394.54)	180.67 (40.95-313.17)	×2.34	0.43
HGF	534.72 (210.44)	497.91 (375.67-679.67)	231.23 (114.08)	204.03 (156.05-258.75)	×0.51	≤0.001
PDGF-AA	25462.54 (13742.23)	23429.45 (16404.90-32176.13)	132725.23 (53608.77)	137269.89 (90062.55-183956.91)	×6.19	≤0.001
PDGF-BB	9070.09 (6484.37)	6807.38 (5424.22-13513.71)	49143.68 (18068.74)	51779.04 (29826.86-62573.63)	×6.43	≤0.001

**Table 4:** Differences in inflammatory cytokine content between serum and platelet-rich plasma (PRP). Values (pg/ml) are presented as arithmetic mean (standard deviation) and median (Q1-Q3). The ability to concentrate the cytokines in PRP vs. serum is shown as the “ratio.” The significance of the comparison is presented as *p* value.

	Serum		PRP		Ratio	*p*
	Mean (SD)	Median (Q1-Q3)	Mean (SD)	Median (Q1-Q3)		
IL-1*β*	34.09 (48.04)	18.12 (18.12-19.78)	67.09 (58.67)	43.24 (30.36-82.03)	×2.99	0.002
IFN-*α*2	16.90 (2.54)	16.16 (16.16-16.16)	39.0 (28.68)	26.38 (16.16-50.14)	×2.40	≤0.001
IFN-*γ*	4.98 (0.56)	4.87 (4.87-4.87)	6.66 (3.09)	4.87 (4.87-7.13)	×1.39	0.03
TNF-*α*	18.50 (7.45)	13.17 (13.02-20.98)	33.1 (27.09)	23.56 (13.02-42.72)	×2.12	0.02
MCP-1	140.22 (162.49)	80.3 (30.82-194.90)	107.90 (66.07)	88.71 (63.97-148.27)	×2.13	0.99
IL-6	13.14 (2.61)	12.16 (12.16-12.50)	18.70 (9.77)	14.64 (12.16-22.04)	×1.52	0.009
IL-8	47.45 (96.35)	16.39 (12.14-33.46)	125.99 (131.44)	64.38 (46.77-157.73)	×5.89	≤0.001
IL-10	13.62 (4.09)	11.76 (11.76-12.59)	18.84 (8.12)	15.81 (11.76-23.69)	×1.46	0.007
IL-12p70	13.89 (4.22)	12.01 (10.66-17.21)	20.75 (11.30)	16.12 (10.66-27.8)	×1.62	0.01
IL-17A	2.50 (0.87)	1.94 (1.94-2.85)	3.18 (1.69)	2.56 (1.94-4.28)	×1.41	0.15
IL-18	251.82 (167.62)	189.23 (151.49-308.10)	403.01 (292.19)	323.65 (183.26-507.57)	×2.45	0.14
IL-33	117.58 (46.18)	101.795 (83.24-143.64)	176.58 (99.23)	159.09 (95.95-229.86)	×1.75	0.11

## Data Availability

The data used to support the findings of this study are available from the corresponding author upon reasonable request.

## Supplementary Materials

Supplementary Materials Supplementary Table 1: differences between males and females according to age, BMI, and biologically active compounds in whole blood, serum, and PRP. Values are presented as arithmetic mean (standard deviation) and median (Q1-Q3). The significance of the comparison is shown as *p* value.

## References

[1] Hussain N., Johal H., Bhandari M. (2017). An evidence-based evaluation on the use of platelet rich plasma in orthopedics - a review of the literature. *Sicot-J*.

[2] Man D., Plosker H., Winland-Brown J. E. (2001). The use of autologous platelet-rich plasma (platelet gel) and autologous platelet-poor plasma (fibrin glue) in cosmetic surgery. *Plastic and Reconstructive Surgery*.

[3] Tietze D. C., Geissler K., Borchers J. (2014). The effects of platelet-rich plasma in the treatment of large-joint osteoarthritis: a systematic review. *The Physician and Sportsmedicine*.

[4] Chellamuthu G., Muthu S., Khanna M., Khanna V. (2021). ‘Platelet-rich plasma holds promise in management of rheumatoid arthritis’—systematic review. *Rheumatology International*.

[5] Badsha H., Harifi G., Murrell W. D. (2020). Platelet rich plasma for treatment of rheumatoid arthritis: case series and review of literature. *Case Reports in Rheumatology*.

[6] Yan S., Yang B., Shang C. (2016). Platelet-rich plasma promotes the migration and invasion of synovial fibroblasts in patients with rheumatoid arthritis. *Molecular Medicine Reports*.

[7] Tong S., Zhang C., Liu J. (2017). Platelet-rich plasma exhibits beneficial effects for rheumatoid arthritis mice by suppressing inflammatory factors. *Molecular Medicine Reports*.

[8] Oudelaar B. W., Peerbooms J. C., Huis In ’t Veld R., Vochteloo A. J. (2019). Concentrations of blood components in commercial platelet-rich plasma separation systems: a review of the literature. *The American Journal of Sports Medicine*.

[9] Sánchez-González D. J., Méndez-Bolaina E., Trejo-Bahena N. I. (2012). Platelet-rich plasma peptides: key for regeneration. *International Journal of Peptide*.

[10] Werner S., Grose R. (2003). Regulation of wound healing by growth factors and cytokines. *Physiological Reviews*.

[11] Dejnek M., Moreira H., Płaczkowska S. (2021). Analysis and comparison of autologous platelet-rich plasma preparation systems used in the treatment of enthesopathies: a preliminary study. *Advances in Clinical and Experimental Medicine*.

[12] Evanson R., Guyton M. K., Oliver D. L. (2014). Gender and age differences in growth factor concentrations from platelet-rich plasma in adults. *Military Medicine*.

[13] Jayaram P., Yeh P., Patel S. J. (2019). Effects of aspirin on growth factor release from freshly isolated leukocyte-rich platelet-rich plasma in healthy men: a prospective fixed-sequence controlled laboratory study. *The American Journal of Sports Medicine*.

[14] Weibrich G., Kleis W. K. G., Hafner G., Hitzler W. E. (2002). Growth factor levels in platelet-rich plasma and correlations with donor age, sex, and platelet count. *Journal of Cranio-Maxillofacial Surgery*.

[15] Zimmermann R., Arnold D., Strasser E. (2003). Sample preparation technique and white cell content influence the detectable levels of growth factors in platelet concentrates. *Vox Sanguinis*.

[16] Dohan Ehrenfest D. M., Rasmusson L., Albrektsson T. (2009). Classification of platelet concentrates: from pure platelet-rich plasma (P-PRP) to leucocyte- and platelet-rich fibrin (L-PRF). *Trends in Biotechnology*.

[17] Dhurat R., Sukesh M. (2014). Principles and methods of preparation of platelet-rich plasma: a review and author′s perspective. *Journal of Cutaneous and Aesthetic Surgery*.

[18] Lana J. F. S. D., Purita J., Paulus C. (2017). Contributions for classification of platelet rich plasma - proposal of a new classification: MARSPILL. *Regenerative Medicine*.

[19] Marx R. E. (2001). Platelet-rich plasma (PRP): what is PRP and what is not PRP?. *Implant Dentistry*.

[20] Mishra A., Pavelko T. (2006). Treatment of chronic elbow tendinosis with buffered platelet-rich plasma. *The American Journal of Sports Medicine*.

[21] Mazzucco L., Balbo V., Cattana E., Guaschino R., Borzini P. (2009). Not every PRP-gel is born equal Evaluation of growth factor availability for tissues through four PRP-gel preparations: Fibrinet®, RegenPRP-Kit®, Plateltex® and one manual procedure. *Vox Sanguinis*.

[22] Kobayashi Y., Saita Y., Nishio H. (2016). Leukocyte concentration and composition in platelet-rich plasma (PRP) influences the growth factor and protease concentrations. *Journal of Orthopaedic Science*.

[23] Cieślik-Bielecka A., Bold T., Ziółkowski G., Pierchała M., Królikowska A., Reichert P. (2018). Antibacterial activity of leukocyte- and platelet-rich plasma: an in vitro study. *BioMed Research International*.

[24] Zhang L., Chen S., Chang P. (2016). Harmful effects of leukocyte-rich platelet-rich plasma on rabbit tendon stem cells in vitro. *The American Journal of Sports Medicine*.

[25] Sundman E. A., Cole B. J., Fortier L. A. (2011). Growth factor and catabolic cytokine concentrations are influenced by the cellular composition of platelet-rich plasma. *The American Journal of Sports Medicine*.

[26] Oh J. H., Kim W. O. O., Park K. U., Roh Y. H. (2015). Comparison of the cellular composition and cytokine-release kinetics of various platelet-rich plasma preparations. *The American Journal of Sports Medicine*.

[27] Magalon J., Bausset O., Serratrice N. (2014). Characterization and comparison of 5 platelet-rich plasma preparations in a single-donor model. *Arthroscopy: The Journal of Arthroscopic & Related Surgery*.

[28] Castillo T. N., Pouliot M. A., Kim H. J., Dragoo J. L. (2011). Comparison of growth factor and platelet concentration from commercial platelet-rich plasma separation systems. *The American Journal of Sports Medicine*.

[29] Khan S. A., Joyce J., Tsuda T. (2012). Quantification of active and total transforming growth factor-*β* levels in serum and solid organ tissues by bioassay. *BMC Research Notes*.

[30] Molloy T., Wang Y., Murrell G. A. C. (2003). The roles of growth factors in tendon and ligament healing. *Sports Medicine*.

[31] Dragoo J. L., Korotkova T., Wasterlain A. S., Pouliot M. A., Kim H. J., Golish S. R. (2012). Age-related changes of chondrogenic growth factors in platelet-rich plasma. *Operative Techniques in Orthopaedics*.

[32] Lippross S., Moeller B., Haas H. (2011). Intraarticular injection of platelet-rich plasma reduces inflammation in a pig model of rheumatoid arthritis of the knee joint. *Arthritis and Rheumatism*.

[33] Wang W., Liu J., Yang B. (2017). Modulation of platelet-derived microparticles to adhesion and motility of human rheumatoid arthritis fibroblast-like synoviocytes. *PLoS One*.

[34] Saif D. S., Hegazy N. N., Zahran E. S. (2020). Evaluating the efficacy of intra-articular injections of platelet rich plasma (PRP) in rheumatoid arthritis patients and its impact on inflammatory cytokines, disease activity and quality of life. *Current Rheumatology Reviews*.

