# Supplementary material for: Leukocyte-Rich Platelet-Rich Plasma as an Effective Source of Molecules That Modulate Local Immune and Inflammatory Cell Responses
Source: Oxid Med Cell Longev. 2022 Aug 2;2022:8059622. doi: 10.1155/2022/8059622 (PMC9363181; doi:10.1155/2022/8059622)
Supplement: Supplementary Materials — Supplementary Table 1: differences between males and females according to age, BMI, and biologically active compounds in whole blood, serum, and PRP. Values are presented as arithmetic mean (standard deviation) and median (Q1-Q3). The significance of the comparison is shown as p value. [file 8059622.f1.docx]

Supplementary Table 1. Differences between males and females according to age, BMI and biologically active compounds in whole blood, serum and PRP. Values are presented as arithmetic mean (standard deviation) and median (Q1-Q3). The significance of the comparison is shown as *p-*value.

|  | **Females** | | **Males** | |  |
| --- | --- | --- | --- | --- | --- |
|  | **Mean (SD)** | **Median (Q1 – Q2)** | **Mean (SD)** | **Median (Q1 – Q2)** | **p** |
| Age [years] | 49.69 (4.41) | 50 (46.5 – 52) | 48.47 (7.5) | 50 (44 – 55) | 0.582 |
| BMI | 26.1 (5.03) | 25.08 (22.35 – 28.63) | 28.68 (4.12) | 27.34 (25.44 – 30.64) | 0.130 |
|  |  |  |  |  |  |
| **PRP** | | | | | |
| WBC [10^3^/μl] | 25.83 (7.15) | 24.72 (21.05 – 31.36) | 34.64 (9.96) | 35.38 (24.7 – 45.23) | < 0.05 |
| Neutrophiles [10^3^/μl] | 10.79 (5.77) | 11.39 (6.83 – 13.66) | 14.77 (7.11) | 18.03 (10.2 – 20.25) | 0.096 |
| Lymphocytes [10^3^/μl] | 12.34 (4.19) | 11.82 (9.73 – 13.72) | 16.13 (5.27) | 14.75 (12.03 – 21.08) | < 0.05 |
| Monocytes [10^3^/μl] | 2.32 (0.71) | 2.25 (1.96 – 2.89) | 3.34 (1.16) | 3.31 (2.71 – 4.37) | 0.006 |
| Eosinophiles [10^3^/μl] | 0.13 (0.16) | 0.06 (0.03 – 0.18) | 0.22 (0.16) | 0.21 (0.05 – 0.31) | 0.069 |
| Basophiles [10^3^/μl] | 0.2 (0.15) | 0.14 (0.1 – 0.31) | 0.18 (0.09) | 0.18 (0.11 – 0.26) | 0.984 |
| RBC [10^6^/μl] | 0.95 (0.52) | 0.74 (0.62 – 1.02) | 0.89 (0.46) | 0.67 (0.52 – 1.23) | 0.520 |
| PLT [10^3^/μl] | 1108.5 (598.81) | 1214.5 (515 – 1476) | 1057.6 (369.26) | 1032 (816 – 1296) | 0.780 |
| TGF-β1, free active [pg/ml] | 392.45 (253.91) | 336.5 (237.3 – 451.23) | 373.6 (257.95) | 343.26 (257.9 – 394.29) | 0.890 |
| EGF [pg/ml] | 233.68 (134.68) | 244.3 (101.51 – 312.78) | 254.25 (177.58) | 206.71 (116.43 – 404.62) | 0.934 |
| FGF-basic [pg/ml] | 324.55 (159.75) | 281.62 (225.27 – 397.97) | 1198.18 (2922.48) | 364.19 (299.8 – 428.55) | 0.156 |
| VEGF [pg/ml] | 254.85 (389.29) | 98.185 (38.54 – 287.08) | 404.16 (399.48) | 225.79 (83.68 – 683.32) | 0.205 |
| HGF [pg/ml] | 228.21 (105.85) | 213.93 (168.02 – 249.66) | 234.46 (126.26) | 203.45 (154.82 – 290.69) | 0.983 |
| PDGF-AA [pg/ml] | 131264.78 (53824.16) | 141055.15 (91260.69 – 181068.92) | 134283.04 (55220.91) | 132774.2 (90062.55 – 193193.8) | 0.879 |
| PDGF-BB [pg/ml] | 47025.82 (17870.22) | 50207.32 (31570.24 – 60480.01) | 51402.72 (18623.90) | 53419.26 (29826.86 – 62932.69) | 0.510 |
| IL-1β [pg/ml] | 39.49 (21.41) | 39.9 (19.83 – 49.41) | 98.63 (71.71) | 75.64 (34.73 – 151.71) | < 0.05 |
| IFN-α2 [pg/ml] | 27.78 (14.36) | 22.59 (16.16 – 39.39) | 50.96 (35.29) | 47.88 (20.21 – 77.7) | < 0.05 |
| IFN-γ [pg/ml] | 5.65 (1.75) | 4.87 (4.87 – 5.7) | 7.81 (3.89) | 6.39 (4.87 – 8.72) | 0.101 |
| TNF-α [pg/ml] | 23.74 (13.65) | 19.04 (13.02 – 31.76) | 43.07 (34.15) | 28.28 (13.54 – 69.04) | 0.118 |
| MCP-1 [pg/ml] | 101.53 (57.44) | 81.01 (65.57 – 136.93) | 115.17 (76.32) | 94.22 (63.97 – 151.18) | 0.632 |
| IL-6 [pg/ml] | 15.81 (4.4) | 14.49 (12.16 – 18.75) | 21.99 (12.99) | 16.01 (12.16 – 25.01) | 0.271 |
| IL-8 [pg/ml] | 69.74 (52.34) | 50.61 (43.55 – 82.66) | 185.98 (163.13) | 119.31 (51.99 – 316.42) | < 0.05 |
| IL-10 [pg/ml] | 14.7 (3.09) | 14.11 (11.76 – 17.28) | 23.56 (9.53) | 24.34 (13.48 – 28.2) | < 0.05 |
| IL-12p70 [pg/ml] | 15.17 (4.69) | 14.66 (10.66 – 18.2) | 26.7 (13.29) | 27.8 (13.93 – 34.35) | < 0.05 |
| IL-17A [pg/ml] | 2.45 (0.99) | 1.94 (1.94 – 2.61) | 3.96 (1.95) | 3.93 (1.94 – 4.74) | < 0.05 |
| IL-18 [pg/ml] | 251.77 (141.57) | 194.32 (155.87 – 334.12) | 564.33 (327.8) | 492.4 (296.32 – 959.07) | < 0.05 |
| IL-33 [pg/ml] | 128.97 (46.14) | 112.15 (90 – 161.02) | 227.37 (116.04) | 224.97 (107.24 – 276.7) | < 0.05 |
| **Whole blood** | | | | | |
| WBC [10^3^/μl] | 6.78 (1.06) | 6.97 (5.72 – 7.78) | 6.42 (1.6) | 6.62 (4.61 – 7.74) | 0.466 |
| Neutrophiles [10^3^/μl] | 4.34 (1.09) | 4.14 (3.21 – 5.3) | 3.71 (1.29) | 3.96 (2.44 – 4.94) | 0.153 |
| Lymphocytes [10^3^/μl] | 1.87 (0.51) | 1.73 (1.64 – 1.98) | 2.06 (0.52) | 1.87 (1.55 – 2.59) | 0.350 |
| Monocytes [10^3^/μl] | 0.36 (0.09) | 0.34 (0.29 – 0.44) | 0.43 (0.11) | 0.43 (0.35 – 0.51) | 0.063 |
| Eosinophiles [10^3^/μl] | 0.11 (0.06) | 0.1 (0.07 – 0.13) | 0.2 (0.14) | 0.16 (0.09 – 0.24) | < 0.05 |
| Basophiles [10^3^/μl] | 0.03 (0.02) | 0.03 (0.02 – 0.04) | 0.03 (0.01) | 0.03 (0.02 – 0.04) | 0.900 |
| RBC [10^6^/μl] | 4.56 (0.37) | 4.52 (4.38 – 4.81) | 5.15 (0.28) | 5.18 (4.89 – 5.42) | ≤ 0.001 |
| PLT [10^3^/μl] | 270.8 (66.39) | 270 (238 – 312) | 235.73 (47.27) | 225 (196 – 292) | 0.107 |
| **Serum** | | | | | |
| TGF-β1, free active [pg/ml] | 144.37 (88.93) | 130.21 (57.59 – 236.84) | 159.5 (51.46) | 167.12 (126.14 – 198.91) | 0.583 |
| EGF [pg/ml] | 118.26 (52.13) | 105.59 (72.08 – 159.2) | 100.55 (34.34) | 94.72 (78.05 – 130.18) | 0.306 |
| FGF-basic [pg/ml] | 1065.19 (623.24) | 773.2 (484.02 – 1720.4) | 1125.0 (301.76) | 1170.44 (967.45 – 1333.44) | 0.570 |
| VEGF [pg/ml] | 141.68 (50.55) | 154.04 (105.41 – 173.25) | 165.8 (54.58) | 151.81 (139.14 – 168.69) | 0.612 |
| HGF [pg/ml] | 559.05 (260.44) | 588.25 (321.61 – 739.51) | 506.64 (137.52) | 493.59 (394.27 – 557.95) | 0.521 |
| PDGF-AA [pg/ml] | 25252.81 (13074.01) | 23429.45 (18473.94 – 30788.92) | 25687.25 (14918.73) | 24049.49 (13143.73 – 32235.4) | 0.934 |
| PDGF-BB [pg/ml] | 8840.36 (5556.49) | 7291.22 (6157.65 – 13513.71) | 9316.23 (7562.22) | 6020.43 (4208.4 – 144.08.71) | 0.848 |
|  |  |  |  |  |  |
| IL-1β [pg/ml] | 33.0 (46.04) | 18.12 (18.12 – 18.12) | 35.34 (52.13) | 18.12 (18.12 -20.95) | 0.357 |
| IFN-α2 [pg/ml] | 16.35 (0.75) | 16.16 (16.16 – 16.16) | 17.52 (3.62) | 16.16 (16.16 – 16.16) | 0.461 |
| IFN-γ [pg/ml] | 4.87 (1.1) | 4.87 (4.87 – 4.87) | 5.08 (0.79) | 4.87 (4.87 – 4.87) | 0.765 |
| TNF-α [pg/ml] | 16.99 (6.41) | 13.02 (13.02 – 19.82) | 20.25 (8.4) | 18.59 (13.02 – 30.57) | 0.434 |
| MCP-1 [pg/ml] | 169.66 (202.77) | 75.31 (29.66 – 243.04) | 106.25 (95.43) | 82.92 (31.97 – 132.87) | 0.854 |
| IL-6 [pg/ml] | 13.54 (3.42) | 12.16 12.16 – 12.16) | 12.67 (1.12) | 12.16 (12.16 – 12.83) | 0.782 |
| IL-8 [pg/ml] | 43.12 (84.09) | 15.1 (12.14 – 35.97) | 52.44 (112.2) | 19.2 (12.14 – 30.94) | 0.981 |
| IL-10 [pg/ml] | 12.53 (1.68) | 11.76 (11.76 -12.42) | 14.71 (5.42) | 11.76 (11.76 – 15.19) | 0.629 |
| IL-12p70 [pg/ml] | 13.43 (3.03) | 12.23 (10.66 – 15.35) | 14.38 (5.28) | 10.66 (10.66 – 17.35) | 0.777 |
| IL-17A [pg/ml] | 2.57 (0.83) | 2.04 (1.94 – 3.01) | 2.43 (0.94) | 1.94 (1.94 – 2.43) | 0.266 |
| IL-18 [pg/ml] | 200.66 (135.43) | 165.39 (117.9 – 254.34) | 315.77 (187.17) | 281.27 (185.84 – 437.73) | 0.060 |
| IL-33 [pg/ml] | 126.21 (51.97) | 103.81 (83.24 – 160.89) | 108.95 (39.59) | 92.635 (83.24 – 111.78) | 0.435 |
